# A255 COMMUNICATING NEEDS AND FEATURES OF IBD EXPERIENCES (CONFIDE) SURVEY: PATIENT PERSPECTIVES ON EXPERIENCE OF ULCERATIVE COLITIS SYMPTOMS IN CANADA

**DOI:** 10.1093/jcag/gwad061.255

**Published:** 2024-02-14

**Authors:** J Glass, R Panaccione, t Bessissow, T Hunter Gibble, C Atkinson, M Braun, H Ellis, T Dewar, V Jairath

**Affiliations:** Eli Lilly and Company Canada, Toronto, ON, Canada; University of Calgary Cumming School of Medicine, Calgary, AB, Canada; Division of Gastroenterology, Montreal General Hospital, McGill University Health Center, Montreal, QC, Canada; Eli Lilly and Company, Indianapolis, IN; Adelphi Real World, Bollington, Cheshire East, United Kingdom; Eli Lilly and Company Canada, Toronto, ON, Canada; Adelphi Real World, Bollington, Cheshire East, United Kingdom; Adelphi Real World, Bollington, Cheshire East, United Kingdom; Western University & London Health Sciences Centre, London, ON, Canada

## Abstract

**Background:**

Moderate-to-severe ulcerative colitis (UC) can be associated with impairment in quality of life (Kawalec, 2016).

**Aims:**

The Communicating Needs and Features of IBD Experiences (CONFIDE) study aims to increase understanding of patients’ experiences and the impact of IBD on their lives in the United States, Europe, Japan, and Canada. These data focus on Canadian patients.

**Methods:**

An online, quantitative, cross-sectional survey was conducted (panel recruitment) between February and March 2023 in patients diagnosed with active moderate-to-severe UC in Canada. Moderate-to-severe UC was defined using criteria based on previous treatment, steroid use, and/or hospitalization. Data collected included patient perspectives on their experiences with UC, including symptom burden. Results were summarised descriptively.

**Results:**

The survey was completed by 82 (of 373 contacted – 22% response) patients (65% male, mean age 43.5 years (SD 12.6), mean time since diagnosis 6.3 years (SD 7.6, range 1-33 years)). The top 3 patient-reported symptoms experienced in the past month were diarrhea (39%, n=32), bowel urgency (BU, 31%, n=25), and fatigue/tiredness (29%, n=24). Overall 73% (n=60/82) were receiving advanced therapies (biologic or novel oral therapy), and BU was currently experienced by 28% (n=17) of these patients. Over half of patients had ever experienced BU (56%, n=46); of these, 48% (n=22) reported doing so at least once a week in the past 3 months. Due to fear/anticipation of bowel urgency related accidents, 68% (n=56) of patients with UC reported wearing diaper/pad/protection at least once in the past 3 months. Of those who had worn diaper/pad/protection, 32% (n=18) reported that they suffered from BU in the past month. Due to fear of BU-related accidents, 31% (n=25) of patients declined participating in work/school, 34% (n=28) declined participating in social events, and 26% (n=21) declined participating in sports/physical exercise in the last 3 months.

**Conclusions:**

Out of the top 3 symptoms reported by patients, BU was the second-most common in patients with moderate-to-severe UC in this study of Canadian patients. A substantial proportion of patients with moderate-to-severe UC continue to report BU, despite receiving advanced therapies; with two-thirds requiring to wear diapers or pads. Further work is needed to understand the pathophysiology of urgency and the efficacy of medical therapies to improve urgency using validated outcome measures.

References

Kawalec P. Indirect costs of inflammatory bowel diseases: Crohn's disease and ulcerative colitis. A systematic review. Arch Med Sci. 2016 Apr 1;12(2):295-302

**Funding Agencies:**

Eli Lilly and Company

